# On the Danger of Mistaking Certain Infantile Affections for Worm-Cases, with Remarks on the Impropriety of Giving Drastic Purges and Strong Anthelmintics

**Published:** 1815-04

**Authors:** J. K. Walker

**Affiliations:** Physician to the Huddersfield Dispensary.


*For the Medical and Physical Journal.*

### *On the Danger of mistaking certain Infantile Affections for Worm-Cases, with Remarks on the Impropriety of giving Drastic Purges and strong Anthelmintics;* by J. K. Walker, M.D.

HAVING been present, some time back, at the *dissectio cadaveris* of a child, æt. four years (a patient of a medical gentleman at a distance from this town), who had been treated with the most drastic anthelmintics with the design of destroying worms, which were supposed to have given rise to the disease, (the patient having once parted with some); I was forcibly reminded of the correctness of Dr. Hunter's opinion, who had dissected great numbers of children, supposed to have died of worm-fevers, whose complaints had been met by a corresponding treatment, and in whom the doctor observed there not only appeared on dissection to be no worms, but visible demonstrations of the disorder arising from a different source.

In the patient here alluded to, symptoms had arisen certainly very closely resembling those incident on cases of actually existing worms. There had been, for instance, great hardness, tension, deep-seated pain of the abdomen, slimy slimy stools, griping pains, thirst and feverishness, fetid breath and fickleness of appetite, and some few worms had once been since. Yet so much had the essential part of the disease been overlooked, that a scrophulous swelling of the submaxillary glands in this patient, and alarming emaciation of the body, did not once suggest the idea of its arising from mesenteric affection; as plainly appeared on inspecting the morbid state of the mesenteric glands, which were thickened and filled with a curdy matter.

Now we know by experience, that, when the diseased affections or indurations of these glands are discovered in their incipient stage, they have often been discussed by appropriate regimen, with the occasional use of small doses of calomel, as Dr. Hamilton and other writers on scrophula have advised, conjoining, at the same time, the use of the warm-bath every night at bed-time, along with medicated friction applied to the belly evening and morning. Possibly some such method of treatment as this might have palliated, if not completely arrested, the disorder. Be this as it may, few, I think, will doubt that the use of drastic vermifuges in this patient, such as male fern, tin filings, oil of turpentine, hellebore, with a farrago of chemical and mechanical antidotes, might annihilate the worms, but would expedite the fate of the unhappy sufferer.

It is well known, that where there is a predisposition or hereditary tendency to scrophula, any long-protracted disease may call it into action, and the most trifling mark of indisposition in such a strumous diathesis, may, by neglect, go on so far as to produce the most alarming scrophulous affections; and, in children, disorders of the chylopoietic viscera are frequently the first forerunners of this proteal malady.

Though it occasionally occurs, as Dr. Darwin remarks, that the mucus of the intestines is so hardened as to line the intestines and to come away in skins, rolled up so as to resemble worms, for which they are frequently mistaken; yet it is very probable, I think, in this case, that worms did actually exist, and, as we know, that they usually produce more or less of colicky pain, with swelling and hardness of belly, &c. they might have been the first source of inducing a morbid state of the mesenteric glands; but, whether they preceded or were synchronous in their origin and progress with the glandular affection, or constituted a part of the disease, no policy, I presume, can be more disastrous than to treat such complication as if it were a simple case of worm fever, a practice too often acted up to, I fear, in the ill-defined diseases of children.

 Cathartics,

Cathartics, skilfully administered, I admit, have been usefully exhibited in mesenteric affections, but such drastic ones as are ordinarily resorted to in worm cases, if often repeated, must infallibly aggravate the symptoms. Etmuller, after enumerating the best remedies for this affection, wisely adds "interpositis *blandis purgantibus*, tractentur tamen (morbi mesenterici) *caute et sine violentia*, exasperantur alias et deteriores evadunt, ob nervos tunc irritatos." In almost any other disease which may arise from, be mistaken for, or be combined with, worms, such as the infantile remittent fever, hydrocephalus, sequelæ of dentition, such active aperients, from the accustomed torpor of the bowels, are fully warranted, as many of the symptoms depend in part on intestinal irritation, or, in some cases, from absorption of their putrid contents.

Wherever, however, there is a suspicion of such a combination as I have above described, or any appearance of scrophulous taint, a prudent practitioner, I think, will defer all violent remedies, and trust to a more lenient, if more protracted, treatment. In such compound cases, those aperients, I contend, are most eligible, *of which a frequent repetition can be borne with the least debilitating tendency.* This hypothesis, I think, will obtain even in most cases of simple worm fevers. Of this sort are purging waters, particularly the sulphureous, especially if they be assisted by the frequent administration of glysters, medicated according to the discretion of the prescriber and the species of worms which infests the bowels.

In upwards of thirty cases of worms which have fallen under my care, this last half year, at the Huddersfield Dispensary, few have not experienced relief, and many the entire removal, of the complaint, without either hellebore fætid, oil of turpentine, tin filings, or the rest of the drastic vermifuges so much in vogue. A continued use of artificial mineral waters, some in imitation of the Harrowgate, others of the Cheltenham, with the occasional use of the spigelia, and sometimes glysters or frictions, simple or medicated, over the abdomen, have succeeded in extirpating the severest cases of lumbricus or ascarides. But, when there is any latent scrophulous taint combined with the worms, as is often the case, it then becomes us to guard against any mesenteric affection, and to use gentle eccoprotics merely. I have found Dr. Butter's plan in giving the extr. conii in a mixture with small doses of sal. polychrest, with the ordinary treatment of mesenteric affections superadded, efficacious in palliating, where it has not effected a cure.

Children, it is remarked, are seldom troubled with worms before before they are weaned, unless the mother herself be affected with them; not so with mesenteric affections invariably, or with hydrocephalus, which attack children from the age of a few months to ten or twelve years or upwards, and are more idiopathic and dangerous the earlier they attack. A mother who suckles her child, if infested with worms, will frequently communicate *per lac.* the disorder to her infant; and if she be at the same time of a strumous taint, her infant sucks in the seeds of two disorders at once.

J. K. WALKER, M.D.

*Physician to the Huddersfield Dispensary.*

